# Killing pain?: a population-based registry study of the use of prescription analgesics, anxiolytics, and hypnotics among all children, adolescents and young adults in Norway from 2004 to 2019

**DOI:** 10.1007/s00787-022-02066-8

**Published:** 2022-08-27

**Authors:** Helle Stangeland, Marte Handal, Svetlana Ondrasova Skurtveit, Helene Flood Aakvaag, Grete Dyb, Tore Wentzel-Larsen, Monica Baumann-Larsen, John Anker Zwart, Kjersti Storheim, Synne Øien Stensland

**Affiliations:** 1 Norwegian Centre for Violence and Traumatic Stress Studies (NKVTS), Gullhaugveien 1, 0484 Oslo, Norway; 2 Division of Clinical Neuroscience, Department of Research and Innovation, Oslo University Hospital, Oslo, Norway; 3 Institute of Clinical Medicine, University of Oslo, Oslo, Norway; 4 Department of Mental Disorders, Norwegian Institute of Public Health, Oslo, Norway; 5 Norwegian Centre for Addiction Research, University of Oslo, Oslo, Norway; 6 Centre for Child and Adolescent Mental Health, Eastern and Southern Norway, Oslo, Norway; 7 Department of Physiotherapy, Oslo Metropolitan University, Oslo, Norway

**Keywords:** Pain, Anxiety, Sleep, Prescription drugs, Pharmaco-epidemiology

## Abstract

The ongoing opioid epidemic has been a global concern for years, increasingly due to its heavy toll on young people’s lives and prospects. Few studies have investigated trends in use of the wider range of drugs prescribed to alleviate pain, psychological distress and insomnia in children, adolescents and young adults. Our aim was to study dispensation as a proxy for use of prescription analgesics, anxiolytics and hypnotics across age groups (0–29 years) and sex over the last 15 years in a large, representative general population. The study used data from a nationwide prescription database, which included information on all drugs dispensed from any pharmacy in Norway from 2004 through 2019. Age-specific trends revealed that the prevalence of use among children and adolescents up to age 14 was consistently low, with the exception of a substantial increase in use of melatonin from age 5. From age 15–29, adolescents and young adults used more prescription drugs with increasing age at all time points, especially analgesics and drugs with higher potential for misuse. Time trends also revealed that children from age 5 were increasingly dispensed melatonin over time, while adolescents from age 15 were increasingly dispensed analgesics, including opioids, gabapentinoids and paracetamol. In contrast, use of benzodiazepines and z-hypnotics slightly declined in young adults over time. Although trends were similar for both sexes, females used more prescription drugs than their male peers overall. The upsurge in use of prescription analgesics, anxiolytics and hypnotics among young people is alarming.

*Trial registration* The study is part of the overarching Killing Pain project. The rationale behind the Killing Pain research was pre-registered through ClinicalTrials.gov on April 7, 2020. Registration number NCT04336605; https://clinicaltrials.gov/ct2/show/record/NCT04336605.

**Supplementary Information:**

The online version contains supplementary material available at 10.1007/s00787-022-02066-8.

## Introduction

Increasing use of prescription drugs for pain, psychological distress and insomnia among young people is a growing global concern [35]. While pharmacological treatment can provide effective short-term symptom relief, such as in acute and palliative care, potential misuse involves risk in terms of negative health consequences. For clinicians working with young patient populations, there is a fine line between undertreating these often co-occurring conditions and limiting potential misuse. However, despite these concerns the scope and trends of use for the wider range of prescription analgesics, anxiolytics and hypnotics have rarely been studied systematically, especially across the entire developmental trajectory and over extended time periods.

Prescription drugs with higher potential for misuse, such as opioids, gabapentinoids, benzodiazepines and z-hypnotics, are generally not recommended for children and adolescents unless they have a clear indication for treatment, which makes adequate systematic surveillance critical [47]. Potential misuse can lead to dependence, reduced efficacy and increased risk of acute and long-term morbidity and mortality [8, 21, 51]. Importantly, symptoms of cognitive impairment and sedation may also negatively impact social participation and school performance [2].

Recently, the long-term effects of alternative drugs with lower misuse potential have also been questioned. Although safer in terms of potential for misuse, frequent use of analgesics, such as paracetamol and non-steroidal anti-inflammatory drugs (NSAIDs), has been linked to increased risk of medication-overuse headache, cardiovascular risk, gastrointestinal bleeding and renal failure [12, 13, 30]. Furthermore, the consequences of prolonged use of sleep aids, such as melatonin and alimemazine, have not yet been systematically reviewed in children and adolescents [10, 53]. Over-reliance on these drugs from an early age may therefore give rise to unforeseen adverse effects over time.

While the use of these prescription drugs has been limited in children so far, there has been an increase in use among adolescents in recent years, particularly girls, which indicates increasing use with age and among females [20]. However, the window of development from adolescence to young adulthood has been somewhat understudied, as pediatric and adult populations are typically divided around 18 years. This critical transitional period thus needs to be further investigated to uncover how these trends continue to develop with age. Pain, psychological distress and insomnia are common in adolescents and young adults and can escalate into more serious, chronic health problems if they are left untreated or treated ineffectively [11, 28, 36].

Norway and other Scandinavian countries have access to nationwide health registries that can give accurate estimates of prescription drug use across all age groups, which makes them helpful for studying how trends develop over time in the general population. The aim of this population-based registry study was to uncover trends in use of prescription analgesics, anxiolytics and hypnotics among all children, adolescents and young adults in Norway from 2004 to 2019. Specifically, the study focused on use in the form of prevalence rates and average drug amounts across age groups, over time and by sex.

## Methods

### Study design

This was a population-based registry study using repeated cross-sectional measurements of use of prescription analgesics, anxiolytics and hypnotics based on aggregated data from the nationwide Norwegian Prescription Database (NorPD). Time trends in use, in the form of annual prevalence rates and average annual drug amounts per user measured as defined daily doses (DDD), were studied over a 15-year period from 2004 to 2019, and across age groups and sex.

### Population and procedure

All individuals aged 0–29 years with a national identity number (equivalent to social security number) were included. This resulted in a total study population of approximately 2.7 million over the course of the study period, and an annual population of 1.727 471–1.938 350 individuals depending on the year (2004–2019). All individuals included received health coverage under the national public health scheme, promoting equitable access to healthcare and limiting financial incentives with regards to choice of prescription drugs.

#### Data source

The NorPD is a registry that includes information on all drugs dispensed from pharmacies to all individuals in outpatient care in Norway since 2004 [19]. The database can be used to study prescription drug use outside of hospital settings and institutional care. Use of non-prescription, over-the-counter drugs, is not recorded in the database. The NorPD includes data on patient-centered information, such as age and sex, and drug-centered information, such as dispensation date, amount dispensed and the Anatomical Therapeutic Chemical (ATC) code, which identifies the drug [56]. The dispensed drug amount is registered as defined daily doses (DDD), which is a standardization for the amount of a specific drug that is recommended as a daily maintenance dose when used for its main indication in adults. DDDs for pediatric populations have not yet been developed, which is a challenge within the field of pediatric pharmaco-epidemiology [25]. However, while DDDs are normally assigned for use in adults, the measure can still be used to conduct overall comparisons of trends in drug use in pediatric populations [57]. For the purposes of the present study, drug amounts were calculated as the average number of DDDs prescribed per user per year.

### Measures

#### Age and sex differences over time

The NorPD registry holds aggregated data categorized by age, sex and calendar year. The study population was divided into 6 age groups representing the developmental stages of early childhood (0–4 years), mid-to-late childhood (5–9 years), early adolescence (10–14 years), mid-to-late adolescence (15–19 years), early young adulthood (20–24 years) and mid-to-late young adulthood (25–29 years). Some individuals included in these age groups partially overlapped from one year to the next depending on their ages, with each of the groups including around 300,000 individuals at any time point (range 258,136–369,017).

The data were also grouped by sex and per calendar year, from 2004 to 2019. Where the number of individual users of a specific drug in an age group for a particular sex was less than 5, the results for the group were not shown for that year to comply with the data privacy law regulations of NorPD. This was only the case for drugs that were used less often among children, such as gabapentinoids, z-hypnotics and for anxiolytics with lower misuse potential.

#### Use of prescription analgesics, anxiolytics and hypnotics

Use of prescription drugs with higher potential for misuse and alternatives with lower misuse potential for pain, psychological distress and insomnia were the main outcomes. Drugs were included in the study if they were commonly used in clinical practice, or if their mechanism of action indicated use for these disorders according to evidence-based guidelines [32, 39, 58]. Table 1 gives an overview of the included drugs and their ATC-codes, as well as information about central drugs that obtained marketing authorization during the study period. The specific products that are included in each of the different drug classes in the ATC system are regularly updated through the Norwegian Pharmaceutical Product Compendium [48].

**Table 1:** Overview of the included prescription drugs

Therapeutic subgroup	Drugs with higher potential for misuse (ATC-code)	Drugs with lower potential for misuse (ATC-code)
Analgesics	Opioids: Opioids (N02A) Gabapentinoids: Gabapentin (N03AX12) Pregabalin (N03AX16)^a^	NSAIDs: Anti-inflammatory and anti-rheumatic products, non-steroids (M01A) Paracetamol: Paracetamol (N02BE01) Paracetamol combinations, excl Psycholeptics (N02BE51)^b^
Anxiolytics	Benzodiazepines: Clonazepam (N03AE01) Benzodiazepine derivatives (N05BA, N05CD)	Other anxiolytics: Hydroxyzine (N05BB01) Buspirone (N05BE01)
Hypnotics	Z-hypnotics: Benzodiazepine related drugs (N05CF)	Other hypnotics: Melatonin (N05CH01)^a, c^ Clomethiazole (N05CM02) Dexmedetomidine (N05CM18) Alimemazine (R06AD01)^c^

^a^Pregabalin (Lyrica) was approved for use in 2004 and melatonin (Circadin) was approved for use in 2012

^b^Paracetamol combinations, excl. psycholeptics included Dolerin (+ ibuprofen), Paracetduo (+ caffeine) and Paralen (+ acetylsalicylic acid, caffeine)

^c^Melatonin and alimemazine were also studied individually

All included drugs were categorized by their potential for misuse. The drugs categorized as having higher potential for misuse listed in Table 1 were all up until 2021 either primarily (1) not recommended for use in children and adolescents outside of hospital settings or institutional care, or (2) recommended only for restricted, short-term use in adults outside of hospital settings or institutional care [47]. The drugs in the category with lower misuse potential are primarily not indicated for long-term use (e.g., paracetamol and NSAIDs) in any age group.

The therapeutic subgroups were analgesics, anxiolytics and hypnotics. Clonazepam, gabapentin and pregabalin were included even though they are listed as anti-epileptics in the ATC-system, because they have higher potential for misuse and are sometimes used to treat generalized anxiety disorder in adults and neuropathic pain, in addition to epilepsy. Clonazepam was listed as a benzodiazepine and gabapentin and pregabalin as analgesics (gabapentinoids), in line with their mechanisms of action. Furthermore, alimemazine was included as a hypnotic despite being classified as an antihistamine, because it has traditionally been used as a sleep aid in the Norwegian pediatric population. Alimemazine and melatonin were also studied individually, as these are the most common hypnotics used for pediatric insomnia. In addition, clinical practice and evidence-based guidelines related to the use of these drugs have changed considerably over time (see Supplementary material).

#### Prevalence and amounts

Dispensation was used as a proxy for actual drug use and the terms ‘use’ and ‘dispensation’ used interchangeably. Use was primarily operationalized as prevalence rates, defined as prevalence per 1000 persons per year within each of the six age groups listed above. Each individual who collected at least one prescription drug from a pharmacy within a given year was considered to be a user that year. Furthermore, the average number of DDDs dispensed per user per year within each age group was used as an estimate of the drug amounts dispensed among users. DDDs were calculated for all the therapeutic subgroups, except for the anxiolytics and hypnotics with lower misuse potential, as these groups included drugs with different mechanisms of action, making meaningful comparisons unfeasible.

### Statistical methods

Prevalence rates were defined per 1000 individuals for each combination of age group, sex and calendar year. Calculations were based on the total number of individuals in the population and the number of individuals who were dispensed at least one prescription drug within each year, for each of the different drug groups. Confidence intervals were computed for the prevalence rates by Blaker’s procedure that gives accurate confidence intervals for rare events [5].

The NorPD operates with annual mean population sizes based on the total population sizes at the start and end of each year; thus, the mean population sizes in the data set were not always integers. To provide reliable confidence intervals, it was checked that rounding the population mean up or down did not appreciably affect the results of the Blaker’s procedure.

All analyses were performed in R (The R Foundation for Statistical Computing, Vienna, Austria) with computation of confidence intervals using the R package BlakerCI.

The study was conducted in accordance with the Reporting of Studies Conducted Using Observational Routinely Collected Health Data Statement for Pharmacoepidemiology (RECORD-PE) guidelines, which build on the Strengthening the Reporting of Observational Studies in Epidemiology (STROBE) guidelines for pharmaco-epidemiological studies [29].

## Results

### Use of analgesics

**Fig. 1:**
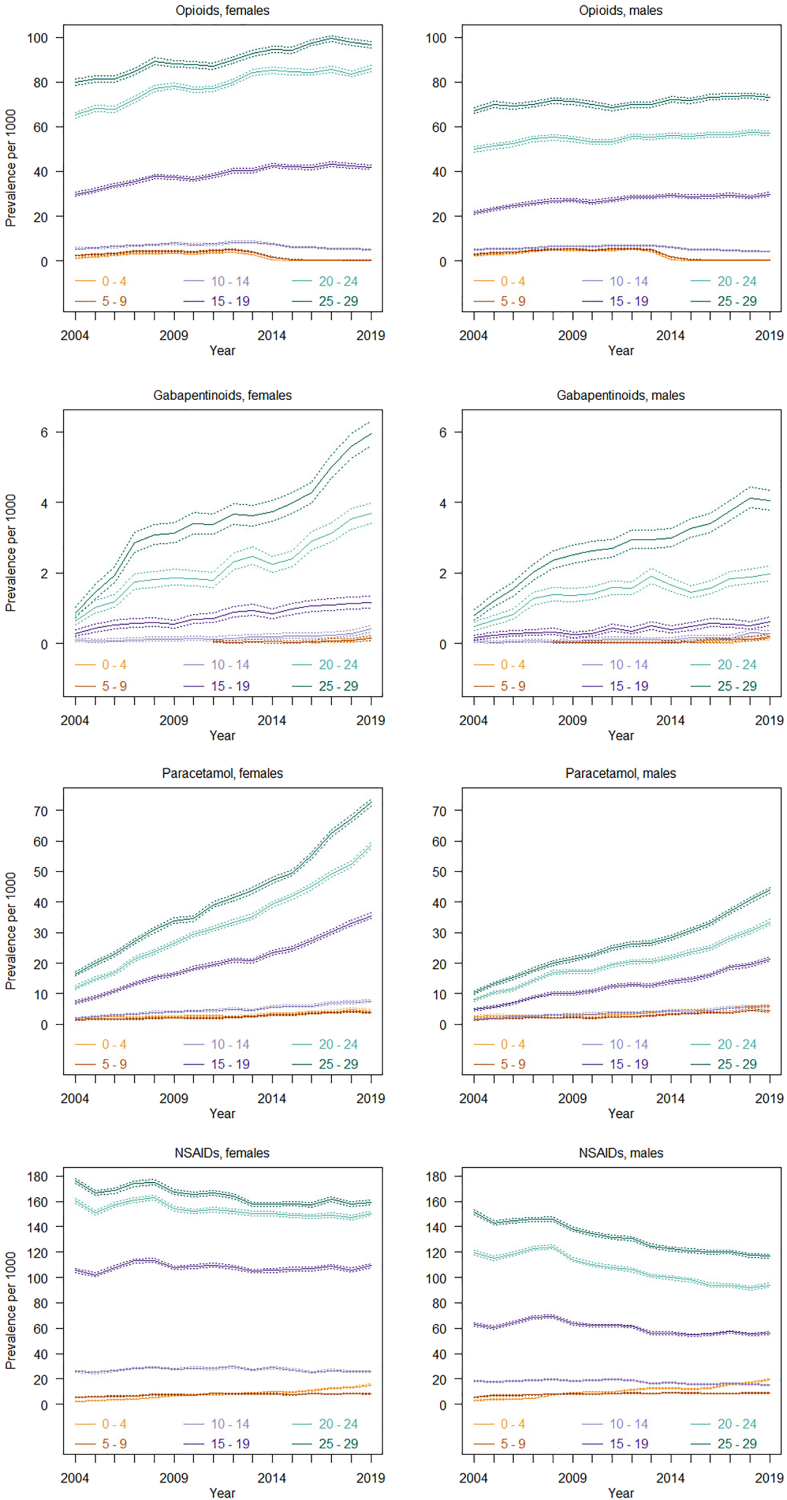
Prevalence of use of analgesics among children, adolescents and young adults from 2004 to 2019

Overall, NSAIDs were the most commonly used analgesic, followed by opioids, paracetamol, and gabapentinoids, respectively (Fig. 1). The results revealed low use of all prescription analgesics in children and adolescents up to age 14 across the study period. However, from ages 15–29, the use increased steadily with age. This age-specific trend was apparent for both sexes, but particularly strong in females. In terms of time trends in the older age groups, the prevalence of use of opioids, gabapentinoids and especially paracetamol increased over the 15-year study period. In addition, the average amounts of paracetamol and NSAIDs dispensed among users increased over time (Supplementary Fig. 1).

### Analgesics with higher potential for misuse: opioids and gabapentinoids

#### Children and younger adolescents (ages 0–14)

Few children and adolescents up to age 14 used opioids (< 0.06%) or gabapentinoids (< 0.05%) in 2019, with results showing consistently low prevalence rates over time (Fig. 1). Additionally, opioid use among children (ages 0–9) declined from 2012 to 2019 (3.8–4.9/1000 in 2012 to 0.1–0.3/1000 in 2019 in females and 5.4–5.5/1000 in 2012 to 0.2–0.4/1000 in 2019 in males).

#### Older adolescents and young adults (ages 15–29)

Among adolescents from age 15, 4.2–9.7% of females and 3–7.3% of males were dispensed opioids, whereas between 0.1 and 0.6% of both sexes were dispensed gabapentinoids in 2019. From 2004, use of opioids (29.7–79.8/1000 in 2004 to 41.9–96.6/1000 in 2019 in females, and 21.5–67.1/1000 in 2004 to 29.7–73/1000 in 2019 in males) and gabapentinoids (0.3–0.9/1000 in 2004 to 1.2–6/1000 in 2019 in females and 0.2–0.8/1000 to 0.6–4.1/1000 in 2019 in males) increased steadily with age and over time. The average amount of opioids dispensed was up to 30 DDDs per user per year and increased with age (Supplementary Fig. 1). Furthermore, adolescents and young adults who were dispensed gabapentinoids used up to 230 DDDs per year on average.

### Analgesics with lower potential for misuse: paracetamol and non-steroidal anti-inflammatory drugs (NSAIDs)

#### Children and younger adolescents (ages 0–14)

Less than 0.8% of children and adolescents up to age 14 used prescription paracetamol and less than 2.6% used prescription NSAIDs in 2019. Trends of use over time were relatively stable, apart from among the youngest children (ages 0–4), for whom use of NSAIDs increased (2.3–5.6/1000 in 2004 to 8.5–15.6/1000 in 2019 in females and 3–5.8/1000 in 2004 to 8.9–19.5/1000 in 2019 in males).

#### Older adolescents and young adults (ages 15–29)

In 2019, 3.6–7.3% of females and 2.1–4.4% of males aged 15 years or older were dispensed paracetamol, while 11–16% of females and 6–12% of males were dispensed NSAIDs. The prevalence of use of prescription paracetamol increased by 3–4 times over the study period (7.2–16.5/1000 in 2004 to 35.5–72.6 in 2019 in females and 4.8–10.5/1000 in 2004 to 21.4–44/1000 in 2019 in males), particularly in females. In contrast, the prevalence of use of prescription NSAIDs declined over the same period among young adults (160.2–176.3/1000 in 2004 to 150.7–159.3/1000 in 2019 in females and 119.6–151.6/1000 in 2004 to 94.1–116.9/1000 in 2019 in males), especially in males. Regarding the amounts, use of prescription paracetamol and NSAIDs increased slightly but steadily over time in adolescents from age 10 (< 50 DDD per user per year).

### Use of anxiolytics

**Fig. 2:**
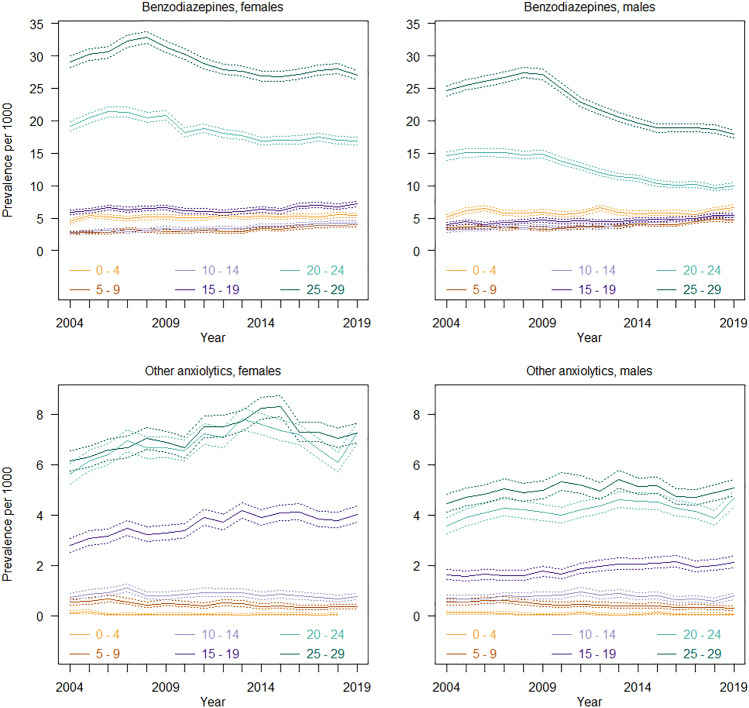
Prevalence of use of anxiolytics among children, adolescents and young adults from 2004 to 2019

Overall, benzodiazepines were more commonly used compared to other anxiolytics (Fig. 2). The results revealed low use of anxiolytics among children and adolescents up to age 14. There was a consistent, gradual increase in prevalence of use with age from 15 years for anxiolytics with lower misuse potential and from 19 years for benzodiazepines. This age-specific trend was particularly strong in females. Over time, both the prevalence rates and amounts of benzodiazepines declined among young adults, but remained relatively stable for the younger age groups (Supplementary Fig. 2).

### Anxiolytics with higher potential for misuse: benzodiazepines

#### Children and adolescents (ages 0–19)

Less than 0.8% of children and adolescents used benzodiazepines in 2019 and the use remained relatively stable across the study period (Fig. 2). In terms of amounts, the average amounts used by children and adolescents aged 5–19 declined slightly over time but did not change considerably (< 80 DDD per user per year), (Supplementary Fig. 2).

#### Young adults (ages 20–29)

In 2019, 1.7–2.7% of young adult females and 1–1.8% of young adult males were dispensed benzodiazepines. The prevalence of use among young adults moderately declined from 2009 (20.8–31.4/1000 in 2009 to 16.9–27/1000 in 2019 in females and 14.8–27.1/1000 in 2009 to 10–18/1000 in 2019 in males), and this trend was especially strong in males. There was a marked decline in the average amounts of benzodiazepines dispensed to young adult males over time (< 220 DDD to < 80 DDD per user per year), but only a slight decline among young adult females.

### Anxiolytics with lower potential for misuse: hydroxyzine and buspirone

#### Children and younger adolescents (ages 0–14)

In 2019, the use of other anxiolytics was very low, less than 0.1% in children and adolescents aged 14 years or younger. The use remained relatively stable over the course of the study period with regard to both prevalence and amounts.

#### Older adolescents and young adults (ages 15–29)

0.4–0.7% of females and 0.2–0.5% of males aged 15 years or older used other anxiolytics in 2019. There was a slight increase in the prevalence of use of other anxiolytics with age and over time (2.8–6.1/1000 in 2004 to 4–7.3/1000 in 2019 in females and 1.6–4.5/1000 in 2004 to 2.1–5.1/1000 in males).

### Use of hypnotics

**Fig. 3:**
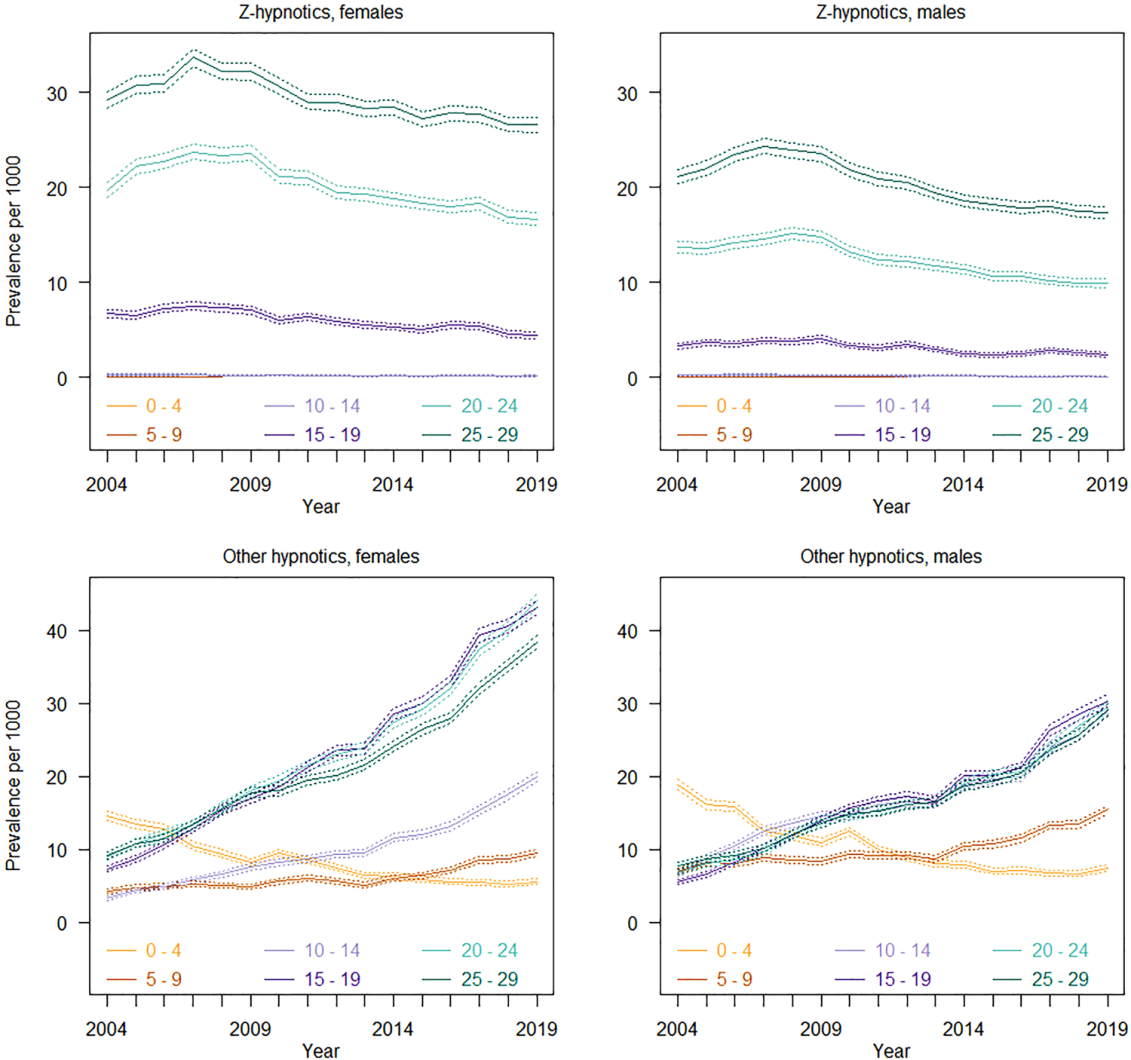
Prevalence of use of hypnotics among children, adolescents and young adults from 2004 to 2019

Overall, the use of z-hypnotics declined over the study period, while the use of hypnotics with lower misuse potential, especially melatonin, increased markedly in children from age 5 (Fig. 3 and Supplementary Figs. 3 and 4). The highest prevalence of melatonin use was observed among adolescents (ages 10–19) and use of z-hypnotics increased with age from age 15–29.

### Hypnotics with higher potential for misuse: z-hypnotics

#### Children and younger adolescents (ages 0–14)

Among children and adolescents up to 14 years, less than 0.1% were prescribed z-hypnotics in 2019, with persistently low rates throughout the study period (Fig. 3).

#### Older adolescents and young adults (ages 15–29)

Approximately 0.4–2.6% of females and 0.2–2.3% of males aged 15 or above were prescribed z-hypnotics in 2019. With regard to age-specific trends, the number of users of z-hypnotics increased consistently with age, in a pattern similar to that observed for both analgesics and anxiolytics. From 2009, there was a slight but steady decline in use of z-hypnotics over time (7.1–32.2/1000 in 2009 to 4.4–26.5/1000 in 2019 in females and 2.4–23.6/1000 in 2009 2.4–17.4/1000 in 2019 in males). The average amounts used were largely stable throughout the study period (< 90 DDD per user per year), except for a slight increase in the youngest adolescent group (ages 10–14), (Supplementary Fig. 3).

### Hypnotics with lower potential for misuse: melatonin, clomethiazole, dexmedetomidine and alimemazine

#### Younger children (ages 0–4)

In 2019, hypnotics with lower potential for misuse were used by less than 0.8% of the youngest children (ages 0–4). Over the study period, a marked decline in use of alimemazine was observed in this group (14.6/1000 in 2004 to 5.6/1000 in 2019 in females and 18.9/1000 in 2004 to 7.4/1000 in 2019 in males), (Supplementary Fig. 4).

#### Older children, adolescents and young adults (ages 5–29)

In 2019, hypnotics with lower misuse potential were used by 1–2% of females and 1.5–2.9% of males aged 5–14, and 3.8–4.4% of females and 2.9–3% of males aged 15 years or older. Over the study period, there was a marked increase in use, mostly related to an increase in use of melatonin among children from age 5. In children and adolescents aged 5–14, more males used hypnotics with lower misuse potential, while from age 15 the prevalence of use was higher in females. Use of alimemazine among females from age 15 also increased with age and over time.

## Discussion

Some important trends were found for the use of prescription analgesics, anxiolytics and hypnotics across age groups, over time and by sex. Among main findings were an increase in overall use with age in adolescents from age 15 at all time points. This age-specific trend particularly applied to the drugs with higher potential for misuse, including opioids, gabapentinoids, benzodiazepines and z-hypnotics, and analgesics with lower misuse potential, including paracetamol and NSAIDs. Use in children and young adolescents up to age 14 was consistently low, except for a substantial increase in use of melatonin from age 5.

From 2004 to 2019, melatonin also became more commonly used among children from age 5, while opioids, gabapentinoids and paracetamol gradually increased among adolescents from age 15. Additionally, the average amounts of paracetamol and NSAIDs increased over the same time period among users. In contrast, use of benzodiazepines and z-hypnotics slightly declined in young adults over time. Although trends were similar for both sexes, females used more drugs compared to their male peers.

### Increasing use of prescription analgesics

The increasing use of prescription analgesics among adolescents and young adults over the last 15 years is perhaps the most concerning finding. Seeing as some analgesics are available over-the-counter (OTC), without a prescription, a possible explanation for this could be a decrease in use of OTC analgesics in favor of prescription analgesics. However, this seems unlikely as sales data indicate that the use of OTC analgesics has been fairly stable across the study period [44]. The increase in use of prescription paracetamol may to some degree result from an intentional shift from prescription of NSAIDs and analgesics with higher potential for misuse, to paracetamol [18]. However, the use of NSAIDs has remained stable and the use of analgesics with higher potential for misuse has increased across the same time period as well. Furthermore, as there is no indication of an increase in acute injuries or other relevant conditions, such as childhood cancer or juvenile arthritis, the increase in prescription analgesics does not seem to be caused by growing numbers of conditions that require treatment with pain medication [9, 34, 45]. Better survival among young people with cancer may, on the other hand, partially explain the observed increase in the prevalence of use of analgesics with higher misuse potential, such as gabapentinoids and opioids [6, 17]. Still, as cancer survivors comprise about 0.5% of the child population and most survivors do not require long-term pain management, this cannot fully explain the overall increase in prescription analgesics use [55]. This leaves the possibility that conditions which do not indicate use of pain medication that seem to be increasing in younger age groups, such as chronic pain or psychological distress, may have contributed to higher use of prescription analgesics [24, 27, 41].

### More restrictive use of prescription anxiolytics and hypnotics with higher potential for misuse

Use of benzodiazepines and z-hypnotics was found to decline in young adults from 2009. The average amounts in users also declined over time, especially among young adult males. Similar trends have been found for z-hypnotics across other Nordic countries, including Denmark and Sweden, although the trends observed in Sweden have been somewhat inconsistent [42, 54]. In Finland, declining trends have been observed for both benzodiazepines and z-hypnotics [50]. This indicates that the increased awareness of these drugs’ potential for misuse may have affected prescription practices. Moreover, doctors may instead be opting for alternatives with lower misuse potential, such as selective serotonin reuptake inhibitors (SSRIs) for psychological distress and melatonin or alimemazine for insomnia.

### Increase in prescription hypnotics with lower potential for misuse

Patterns of use of hypnotics with lower misuse potential in children and adolescents have changed a lot in recent years. Melatonin use was found to increase in children from age 5. Interestingly, children and adolescents between 5 and 14 years used the highest amounts on average (up to 540 DDDs), suggesting regular or even daily use, which is much more than recommended. What is more, because DDDs are calculated for use in adults, this estimate is likely an underestimation. Of note, changes to the marketing authorization of melatonin may have affected prescription practices [15]. However, despite this increase little research has looked into the long-term consequences of melatonin in children and adolescents [53]. This makes this development rather concerning, as melatonin is an exogenous hormone therapy that may affect the body’s own hormone production. Exogenous melatonin could thus have unknown implications when given to children and adolescents that are already going through vast hormonally driven physiological changes related to growth and puberty [4].

### Overall age- and sex-specific trends

The overall use among children and adolescents up to age 14 was much more restrictive compared to the older age groups. Only 2.6% used NSAIDs and less than 1% used any other drug. These consistently low rates are comparable to those found in other countries, including Australia and the United States [3, 37]. From age 15, the number of individuals who used analgesics, as well as anxiolytics and hypnotics with higher potential for misuse increased considerably. While previous studies have found similar trends indicating increasing use with age, the present study sheds new light on how age continuously affects prescription drug use beyond the transition into young adulthood [22, 42, 49].

The findings also reflect the epidemiological sex difference that is well known across studies on pain, depression, insomnia and stress-related disorders (including anxiety and posttraumatic stress disorder). Females 15 years and older were found to use more prescription drugs compared to males, although it is still unclear why these conditions are more prevalent in females from mid-to-late adolescence. While some have argued that this sex difference can best be explained by differences in help-seeking behaviors and level of contact with healthcare providers, others have regarded sex as a biological variable that contributes to important hormonal effects and differential processing of these conditions [31, 40, 46].

### Why are young people using more prescription drugs?

While many factors may contribute to the increase in prescription drug use, the findings ought to be considered in light of societal changes that have developed over the same time period. First, it is possible that young people are experiencing more pain today compared to 15 years ago. If so, the increase in use may be partially explained by changes to young people’s lifestyle, which likely add to overlapping symptoms of pain, psychological distress and insomnia. For example, young people may spend more time on screens and thus sleep less, which can in turn cause them to become more sensitive to pain conditions [23, 43]. The increase in overweight and sedentary lifestyles may also contribute to increased risk of headaches and musculoskeletal pain [1, 7, 52]. Additionally, some evidence indicates that adolescents and young adults are under more stress when it comes to academic performance and that social media use exacerbate symptoms of depression and anxiety [14, 26]. These types of symptoms are currently also worsening among children and adolescents as a consequence of the ongoing COVID-19 pandemic, which supports the need to take their health complaints seriously [38]. Prescription drugs are in addition viewed as safer, easy to access and associated with less societal stigma compared to illicit drugs among adolescents and young adults who misuse [16]. Second, another potential explanation is that it has become more common to prescribe prescription drugs for health complaints to this group.

### Strengths and limitations

The study included the whole Norwegian population and all dispensed drugs from pharmacies across the country. It investigated the entire developmental trajectory, from childhood through to young adulthood, over 15 years. Moreover, it included a wide range of prescription drugs used to treat pain, psychological distress and insomnia. The study also had several limitations. It did not include drugs prescribed through hospitals or institutional settings, nor use of over-the-counter drugs, such as paracetamol, NSAIDs, and (more recently) melatonin, which are readily available without a prescription. Information about why the drugs were prescribed was not available and since the study only included cross-sectional measurements, it was not possible to study individual use over time. Formal statistical tests for time differences were also not feasible since the groups of individuals that were included in consecutive calendar years were not completely independent. It should also be noted that while it is realistic to assume the dispensed drugs were consumed by the recipients, there may have been cases where they were not. Finally, the inferences that can be made from the DDD calculations are limited as DDDs have primarily been developed for adults and are therefore likely less accurate for the actual use in children and young people. DDDs for different products within the same drug group (e.g., opioids) may also vary to some extent and are thus primarily informative for comparisons of overall use of the different drug groups over time [33].

## Conclusion and future directions

Young people from the age of 15 are more likely to use prescription analgesics and drugs with higher potential for misuse with increasing age. This age-specific trend has been consistent over the past 15 years, while use of analgesics has steadily been increasing in this age group over time. During the same time period, use of melatonin for insomnia has also become increasingly common in children from age 5. These trends call for public health interventions and a proactive approach across research and clinical practice to better understand the etiological mechanisms driving the increase in prescription drug use.

## Supplementary Information

Below is the link to the electronic supplementary material.

Supplementary file1 (DOCX 5319 KB)

Supplementary file2 (DOCX 1362 KB)

Supplementary file3 (DOCX 1362 KB)

Supplementary file4 (DOCX 2900 KB)
