# Supplementary material for: Killing pain?: a population-based registry study of the use of prescription analgesics, anxiolytics, and hypnotics among all children, adolescents and young adults in Norway from 2004 to 2019
Source: Eur Child Adolesc Psychiatry. 2022 Aug 27;32(11):2259–70. doi: 10.1007/s00787-022-02066-8 (PMC9419914; doi:10.1007/s00787-022-02066-8)

**SUPPLEMENTARY FIGURE 3**

**Article title:**

**Killing pain? A population-based registry study of the use of prescription analgesics, anxiolytics, and hypnotics among all children, adolescents and young adults in Norway from 2004-2019**

**Author information:**

Helle Stangeland ^1-3^, ORCID: 0000-0001-8609-9926

Marte Handal ^4-5^, ORCID: 0000-0003-1773-0184

Svetlana Ondrasova Skurtveit ^4-5^, ORCID: 0000-0001-7525-9701

Helene Flood Aakvaag ^1^, ORCID: 0000-0003-4869-6038

Grete Dyb ^1,3^, ORCID: 0000-0002-7138-3665

Tore Wentzel-Larsen ^1,6^, ORCID: 0000-0002-0318-4162

Monica Baumann-Larsen ^2-3^, ORCID: 0000-0001-9688-416X

John Anker Zwart ^2-3^, ORCID: 0000-0001-5721-0154

Kjersti Storheim ^2,7^, ORCID: 0000-0002-6887-6901

Synne Øien Stensland ^1-2^, ORCID: 0000-0002-4462-3969

^1^*Norwegian Centre for Violence and Traumatic Stress Studies, Oslo, Norway*

^2^*Department of Research and Innovation, Division of Clinical Neuroscience, Oslo University Hospital, Oslo, Norway*

^3^*Institute of Clinical Medicine, University of Oslo, Oslo, Norway*

^4^*Department of Mental Disorders, Norwegian Institute of Public Health, Oslo, Norway*

^5^*Norwegian Centre for Addiction Research, University of Oslo, Oslo, Norway*

^6^*Centre for Child and Adolescent Mental Health, Eastern and Southern Norway, Oslo, Norway*

^7^*Department of Physiotherapy, Oslo Metropolitan University, Oslo, Norway*

**Corresponding author:**

Helle Stangeland, MSc

Mobile: +47 90029660

Email: h.h.stangeland @nkvts.no


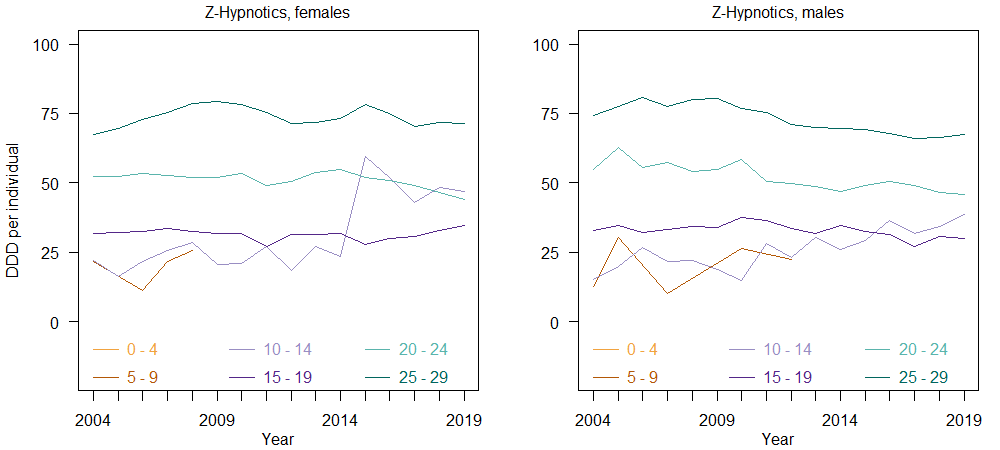

Supplement: Supplementary file 3 — Supplementary file3 (DOCX 1362 KB) [file 787_2022_2066_MOESM3_ESM.docx]
